# Competing Nuclear Quantum Effects and Hydrogen-Bond Jumps in Hydrated Kaolinite

**DOI:** 10.1021/acs.jpclett.2c03896

**Published:** 2023-02-06

**Authors:** Pawan K. J. Kurapothula, Sam Shepherd, David M. Wilkins

**Affiliations:** Centre for Quantum Materials and Technology, School of Mathematics and Physics, Queen’s University Belfast, Belfast BT7 1NN, Northern Ireland, United Kingdom

## Abstract

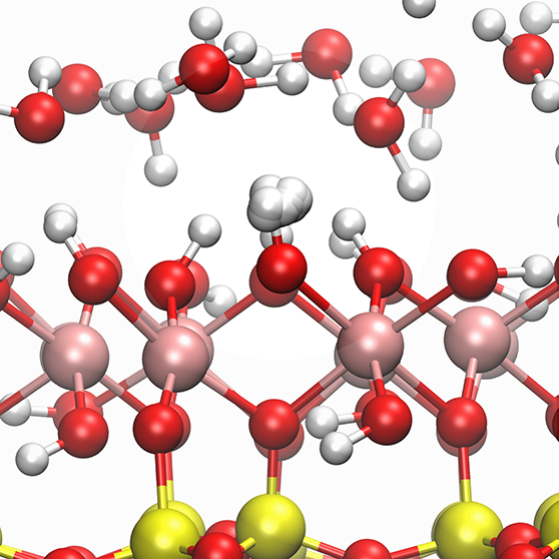

Recent work has shown that the dynamics of hydrogen bonds in pure clays are affected by nuclear quantum fluctuations, with different effects for the hydrogen bonds holding different layers of the clay together and for those within the same layer. At the clay–water interface there is an even wider range of types of hydrogen bond, suggesting that the quantum effects may be yet more varied. We apply classical and thermostated ring polymer molecular dynamics simulations to show that nuclear quantum effects accelerate hydrogen-bond dynamics to varying degrees. By interpreting the results in terms of the extended jump model of hydrogen-bond switching, we can understand the origins of these effects in terms of changes in the quantum kinetic energy of hydrogen atoms during an exchange. We also show that the extended jump mechanism is applicable not only to the hydrogen bonds involving water, but also those internal to the clay.

Hydrogen bonding is central to the properties of clays,^1−4^ with H-bonds not only holding together the layers of clay materials but also controlling their interactions with surrounding solvents such as water. Clay–water systems show a rich variety of hydrogen-bond types and strengths. These interactions are crucial in processes such as the adsorption of solute on the surface of the clay,^5,6^ its wetting,^7−9^ and the swelling of the material by intercalation between the layers.^10,11^ Recent work by the authors^4^ has shown that the properties of dry clays can be affected by nuclear quantum effects (NQEs) such as zero-point energy, leading to weaker hydrogen bonds. The magnitude of these quantum effects depends on whether the H-bonds held together different clay layers or acted within a single layer; in clay–water systems, there is a wider range of H-bonding, with clays accepting H-bonds from the surrounding water, and in some cases able to donate hydrogen bonds to the water. The range of H-bond strengths exhibited by clay–water systems^1−3^ raises the possibility that NQEs may affect different types of hydrogen bond to different degrees.

In this Letter, we use classical and ring polymer molecular dynamics to show that NQEs do indeed affect the hydrogen bonds in clay–water systems to different degrees. By interpreting our results using the angular jump model of hydrogen-bond switching,^12−14^ we are able to understand these quantum effects in terms of the change in confinement of a water molecule as it undergoes a jump. We show further that the angular jump mechanism is present in H-bonds that do not involve water, hinting at its universality.

We focus on NQEs in hydrated kaolinite [Al_2_Si_2_O_5_(OH)_4_], a 1:1-type dioctahedral clay whose layers comprise an octahedral sheet containing aluminum and a tetrahedral sheet containing silicon. Pure kaolinite contains H-bonds both within a clay layer and between neighboring layers; the kaolinite–water interface adds H-bonds donated by water molecules to the silica sheets and H-bonds between water and the aluminol sheets, in which either water or clay O–H groups may be the donor.^2,3^ As in ref (4), since our main focus is on the importance of NQEs, we use the CLAYFF-TRPMD force field developed in that work, which extends the popular CLAYFF force field^15−18^ to give accurate vibrational spectra in path integral simulations. Similarly, the q-TIP4P/F force field was used to model water;^19^ this model accounts well for the experimental properties of pure water in path integral simulations, without requiring a significant computational expense. Classical molecular dynamics (MD), path-integral molecular dynamics (PIMD),^20^ and thermostated ring polymer molecular dynamics (TRPMD)^21^ simulations were carried out using the i-PI^22,23^ and LAMMPS^24^ codes, with NQEs accounted for by the PIMD and TRPMD calculations. To avoid the problem of artificial electric fields at kaolinite–water interfaces described in ref (25), two types of system were simulated: one in which both clay layers were oriented with the aluminol sheets toward the water and one with both silica sheets oriented toward water. Example simulation inputs as well as further details are given in the Supporting Information.

To identify events in which an H-bond donor changed its acceptor, the stable-states picture of chemical reactions was used,^26^ as in refs (12) and (27). That is, a hydrogen-bond switch occurs whenever a donor oxygen O, whose initial acceptor is O_*a*_, forms a hydrogen bond with a new acceptor O_*b*_. We use a strict geometrical criterion for H-bonding, as described in the Supporting Information. The different types of H-bond studied in this letter are given in Figure 1. We began by calculating the hydrogen-bond lifetime correlation functions,^27^
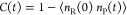
1with the average over all possible H-bonds. *n*_R_(*t*) is 1 if the H-bond is intact at time *t* and 0 otherwise, and *n*_P_(*t*) is 1 if the original H-bond is no longer intact at time *t*, but another H-bond has been formed with the same donor O–H group. Absorbing boundary conditions are used, so that once a hydrogen bond is broken and a new one formed with the same donor, *n*_R_(*t*) = 0 thereafter. By fitting to an exponential decay, *C*(*t*) ≃ e^–*t*/τ_0_^, we find the hydrogen-bond exchange time τ_0_. In path integral simulations the *n*_R_(*t*) and *n*_P_(*t*) functions are evaluated using the ring-polymer centroid.

**Figure 1:**
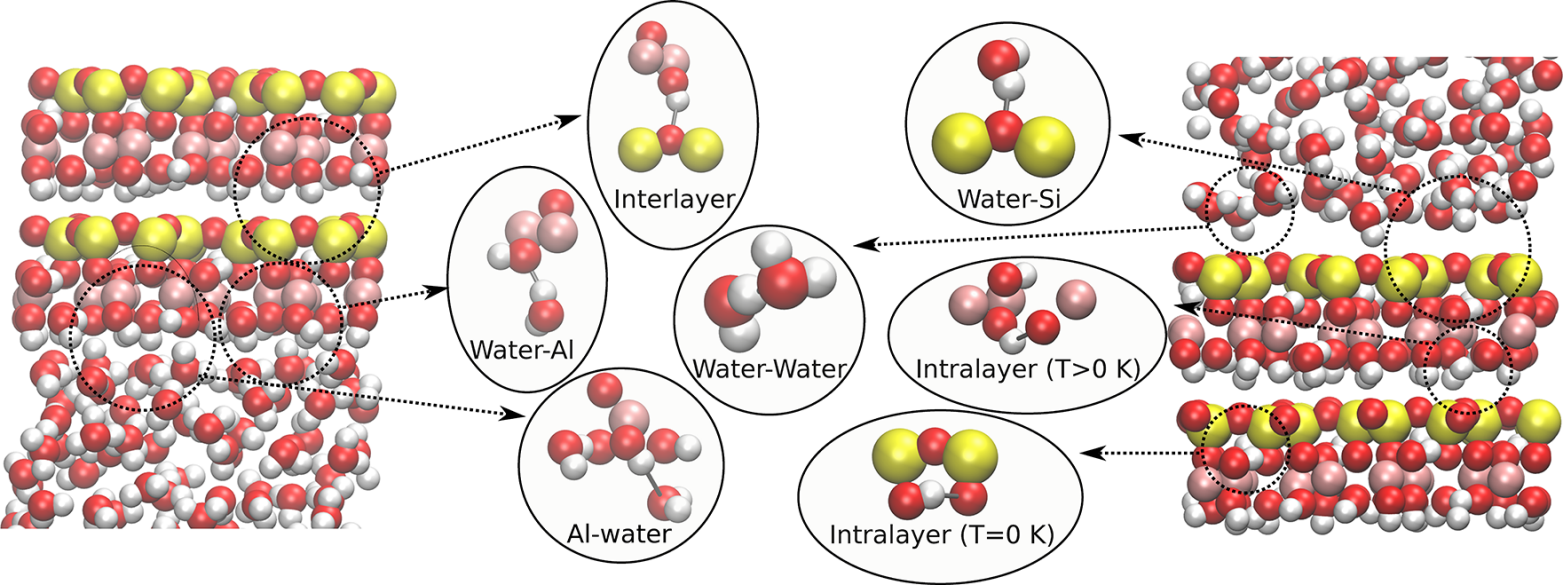
Types of hydrogen bonds considered in this work. The left-hand figure shows the water–alumina surface and the right-hand figure the water–silica surface.

Figure 2 shows the lifetime correlation function *C*(*t*) from MD and TRPMD calculations for the various types of H-bond that are present in these simulations. In Figure 2b, the dynamics of intralayer hydrogen bonds are split into those of H-bonds that remain in the minimum-energy structure (i.e., at zero temperature, denoted *T* = 0 K) and those of H-bonds where the donor would participate in interlayer H-bonds at zero temperature but at finite temperatures is participating in an intralayer H-bond (denoted *T* > 0 K). This distinction was shown to be important in ref (4), in which NQEs on the two types of intralayer hydrogen bonds were shown to be very different. The resulting H-bond exchange times τ_0_ are shown in Table 1 and are in accordance with existing results which show that hydrogen bonding between pairs of water molecules is weaker than with the (hydrophilic) aluminol surface and stronger than with the (hydrophobic) silica surface.^1,2^ The exchange times increase in the order water–silica < water–water < water–aluminol. Table 1 shows that hydrogen bonds donated by aluminol O–H groups and accepted by water are weaker than those donated by water and accepted by the aluminol layer, with the latter being 50% longer lived. Due to the higher electronegativity of Al than of H, the partial negative charge of oxygen in water is greater than that in an aluminol O–H group, making water the stronger H-bond donor. This difference in hydrogen-bonding strength was previously observed in *ab initio* MD simulations.^2,3^ For intralayer H-bonds, there are two distinct time scales: *T* > 0 K H-bonds are much shorter lived than intralayer H-bonds that persist at zero temperature. For the *T* = 0 K intralayer H-bonds, the activation of an exchange involves the rearrangement of the relatively rigid solid structure, meaning that its characteristic time is the longest observed. In Figure 2, some correlation functions are not single exponentials, particularly for water–aluminol and finite-temperature intralayer H-bonds. This behavior has been observed previously for hydrogen-bonding dynamics at water–mineral interfaces, and attributed to a distribution of exchange times τ_0_.^28,29^

**Figure 2:**
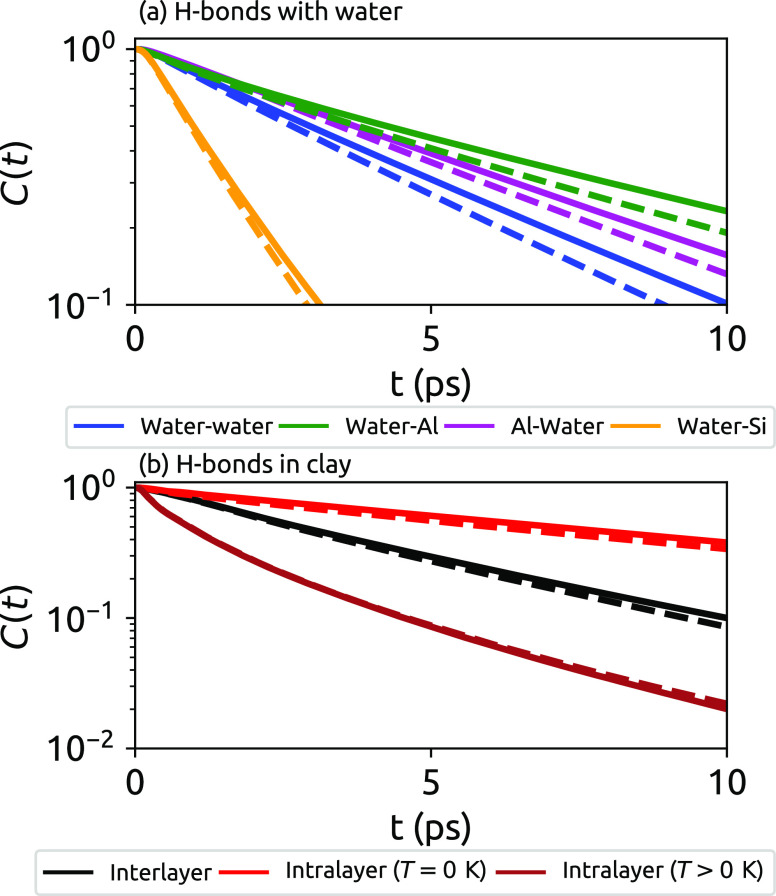
Hydrogen-bond lifetime correlation function *C*(*t*) for (a) water–water H-bonds (blue lines), from water to aluminol (green lines), from aluminol layers to water (magenta lines), and from water to silica (orange lines); (b) interlayer H-bonds (black lines), intralayer H-bonds present in the *T* = 0 K structure (red lines), and intralayer H-bonds that do not exist at *T* = 0 K (brown lines). Solid lines show the results of classical molecular dynamics and dashed lines the results of thermostated ring polymer molecular dynamics.

**Table 1:** Hydrogen-Bond Exchange Times τ_0_ for the Kaolinite–Water System^a^

Type	τ_CL_ (ps)	τ_NQE_ (ps)	τ_CL_/τ_NQE_
Water–water	4.39(4)	3.91(3)	1.12
Water–aluminol	7.3(1)	6.4(1)	1.14
Aluminol–water	5.4(1)	4.87(2)	1.10
Water–silica	1.4(1)	1.3(1)	1.08
Interlayer	4.4(1)	4.15(2)	1.07
Intralayer (*T* = 0 K)	10.6(1)	10.2(3)	1.05
Intralayer (*T* > 0 K)	3.11(4)	3.2(1)	0.97^b^

a For H-bonds involving water, the type is formatted “Donor–acceptor”. Results are shown for classical molecular dynamics (labelled “CL”) and thermostated ring polymer molecular dynamics (labelled “NQE”). The ratio of the two is also shown.

b τ_CL_ and τ_NQE_ are the same within their error bars.

Although all of the quantum effects for H-bond exchange times are small, there is a variation among the different types. For the H-bonds involving water, we note that the longer the classical exchange time τ_CL_, the larger the quantum effect, with water–silica H-bonds having the smallest value for each of these and water–aluminol H-bonds the largest. This is in accord with previous results suggesting that more strongly bound systems, with longer lifetimes, are more susceptible to NQEs, in the context of ion solvation^30,31^ and proton transfer reactions.^32^ Since clays are a significant source of contaminant removal from soil,^6^ this result suggests that NQEs may increase the rate of this process. However, the results for kaolinite’s internal H-bonds do not fit into this trend; although the *T* > 0 K intralayer H-bonds are longer-lived than water–silica H-bonds, there are essentially no NQEs within the error bars. In addition, despite having the same classical exchange time, water–water and interlayer H-bonds incur different quantum effects. Crucially, the differing ratios of classical to quantum exchange times implies that NQEs in clay–water systems cannot be straightforwardly accounted for by running classical simulations at elevated temperatures.

To better understand the different magnitudes of quantum effect seen for hydrated kaolinite we note that the time τ_0_ is a key ingredient in the extended jump model of H-bond switching, in which hydrogen bonds change their acceptors through a large-angle jump of the donor O–H group;^14^ this has been observed in a range of systems,^12,33−35^ including mineral–water interfaces.^36−38^ While it has been shown that water molecules donating hydrogen bonds to a material also change their acceptor via a large-angle jump, it is not yet known whether hydrogen bonds internal to clay minerals also undergo these exchanges, allowing us to investigate the applicability of this mechanism to systems beyond water. Following Laage and Hynes,^27^ we collected examples of events where each kind of H-bond switched and recorded the distance *R*_O–O_*a*__ between the donor O and initial acceptor O_*a*_ and the distance *R*_O–O_*b*__ between the donor and final acceptor O_*b*_, as well as *R*_O_*a*_–O_*b*__ between the initial and final donors, the O_*a*_–O–O_*b*_ angle ϕ, and the angle θ between the O–H bond and the plane bisecting the O_*a*_–O–O_*b*_ angle. For each switching event, the time *t* = 0 was chosen as that when θ = 0.

Figure 3 illustrates the trajectories of *R*_O–O_*a*__, *R*_O–O_*b*__, and θ from classical MD simulations for a selection of types of H-bond, with the remainder of the properties and trajectories for all types of H-bond shown in the Supporting Information. Two points are immediately apparent from this figure: first, while these hydrogen bonds are of different types and have different structures, close to *t* = 0 the angle θ undergoes a rapid jump in all cases; second, unlike the case of H-bonds in pure water, in which there is time-reversal symmetry and the trajectory of *R*_O–O_*a*__ becomes that of *r*_O–O_*b*__ as *t* → −*t*, this symmetry is absent in the kaolinite–water system, wherein an H-bond jump not only leads to the donor changing to a new acceptor but also may lead to a new *type* of acceptor, as when an H atom mediating an H-bond between two O atoms in the same clay layer switches to mediate an H-bond between O atoms of different layers.

**Figure 3:**
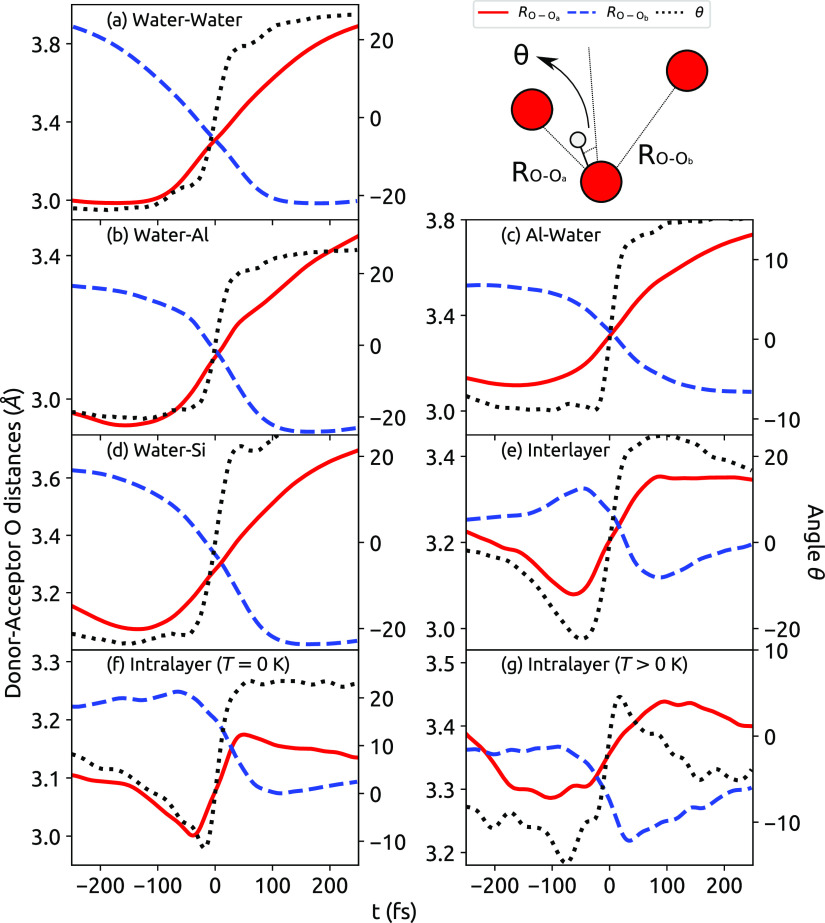
Angular jump trajectories from classical MD calculations. Solid red lines show the distance from the H-bond donor to the initial acceptor, dashed blue lines the distance from the donor to the final acceptor, and dotted black lines the angle θ between the O–H bond and the plane bisecting the vectors from the donor to the initial and final acceptors. The H-bonds are labeled as in Figure 2.

While the trajectories of H-bonds involving water look qualitatively very similar to those observed in pure water,^12,27^ the donor–acceptor distances inside the clay (panels (d)–(f) in Figure 3) behave nonmonotonically, and the behavior of *R*_O–O_*a*__ at *t* ≪ 0 is quite different from *R*_O–O_*b*__ at *t* ≫ 0. This asymmetry reflects the fact that 95% of interlayer H-bonds become intralayer H-bonds after undergoing a jump, and 25% of intralayer H-bonds become interlayer H-bonds. Jumps where both the initial and final acceptor are in the same layer are much more symmetric, as shown in the Supporting Information. This discrepancy may appear at first to be at odds with detailed balance, indicating that jumps do not happen independently—rather, the equilibrium distribution of H-bonds is maintained by reversals of jumps. The populations of interlayer and intralayer H-bonds in dry kaolinite, according to our previous work,^4^ are 0.51:0.19:0.10:0.20 interlayer:*T* = 0 K intralayer:*T* > 0 K intralayer:dangling (i.e., without an H-bond acceptor). The nonmonotonic behavior is less straightforward to understand: future work will focus on investigating whether this is due to the complexity of the mechanism or to limited statistics. The ratios of jumps to different types of H-bond acceptor are also instructive: for water molecules initially donating an H-bond to the aluminol surface, the ratio of final acceptors that are on the aluminol surface to those that are water molecules is around 1:1; for donation to the silica surface, this ratio is closer to 1:3, illustrating the higher strength of H-bonds formed with the aluminol. As shown in the Supporting Information, NQEs make very little difference to the trajectories of H-bond jumps including water, in accord with the results of ref (39).

To understand the origin of these NQEs, we computed the centroid virial kinetic energy tensor,
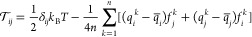
2with *k*_B_ Boltzmann’s constant, *T* the temperature, *q*_*i*_^*k*^ the *i*^th^ Cartesian component of the position of the *k*^th^ ring polymer replica, *f*_*i*_^*k*^ the Cartesian component of the force on this replica, and *n* the number of replicas. As in ref (4), we calculate the component of quantum kinetic energy of H atoms along the O–H bond vector, *r*_OH_ to give , and perpendicular to it, , where .

Figure 4 shows these components, along with the total quantum kinetic energy, close to the transition state of the jump. For H-bonds involving water, the component  parallel to the O–H bond increases at the transition state, indicating that the motion of the H atom becomes more confined in this direction as its H-bonding strength with the initial and final acceptors is relatively low, while  decreases, indicating a greater delocalization in this direction as the O–H bond can move just as easily toward the initial and the final acceptor. The inset of Figure 4 shows this in more detail. Qualitatively, these results are in accord with previous work.^4^ These two effects combine to give the total quantum kinetic energy, which generally decreases at the transition state. Table 2 shows the changes in kinetic energy on going to the transition state, decomposed into parallel and perpendicular components.  does not account for the quantum effect observed by itself, as the average potential energy will also change, but a greater decrease in the reaction barrier due to kinetic energy correlates fairly well with larger quantum effects in τ_0_ (see Table 1).

**Figure 4:**
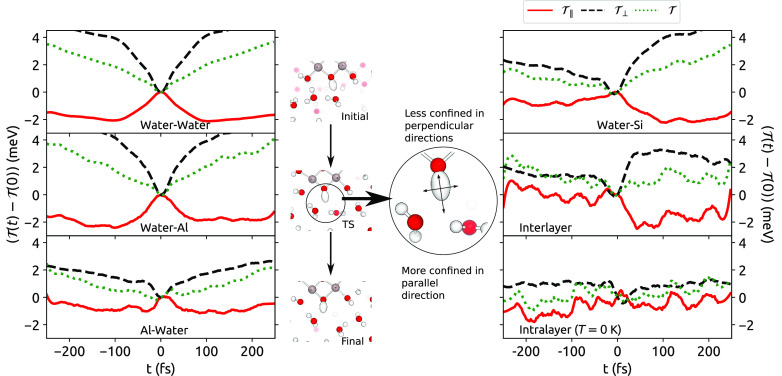
Trajectories for the component of the H atom quantum kinetic energy tensor parallel (, solid red line) and perpendicular (, dashed black line) to the O–H bond during a hydrogen-bond jump. The total kinetic energy is also shown (, dotted green line). In all cases, the differences between the instantaneous value of the tensor element and its value at *t* = 0 are shown. Each panel corresponds to an H-bond type shown in Table 1. The inset illustrates this tensor as an ellipsoid during a jump event: at the transition state, the H atom is able to move toward either the initial or final acceptor, meaning its confinement is low in the perpendicular direction; it is only weakly H-bonded, meaning that it is relatively confined in the parallel direction.

**Table 2:** Change in Quantum Kinetic Energy  (meV) on Going to the Jump Transition State, for H-Bond Jumps^a^

Type		Δ*T*		
Water–water	+2.0	–5.5	–3.5	1.14
Water–aluminol	+2.2	–5.6	–3.4	1.14
Aluminol–water	+0.5	–2.8	–2.3	1.09
Water–silica	+1.7	–4.4	–2.7	1.11
Interlayer	+0.3	–2.0	–1.7	1.07
Intralayer (*T* = 0 K)	+0.6	–1.6	–1.0	1.04

a This is resolved into the component due to motion parallel to the O–H bond () and perpendicular to the O–H bond (). The Boltzmann factor  for each type is also shown, with β = 1/*k*_B_*T* and *T* = 300 K.

A significant cause of the quantum effects in H-bond exchange times is the change in confinement of the H atom, leading to a decrease in the free energy barrier to undergoing a jump. The effects of motion parallel and perpendicular to the O–H bond compete with each other, with the latter dominating in all cases. This competition of effects has been observed before for hydrogen-bonded systems,^19,31,40^ with the dominant effect being determined by the type of motion that contributes most to H-bond breaking.^40^ This means that, while the NQEs in our simulations are extremely subtle, they act as a direct probe of the change in geometry that occurs on going to the transition state. Since only a few hundred jump events were collected for intralayer H-bonds that are only present at finite temperatures, the kinetic energy trajectories are extremely noisy (see the Supporting Information), meaning that the apparent lack of quantum effects in their exchange time τ_0_ cannot yet be fully understood. As in Figure 3, the symmetry in the quantum kinetic energy trajectories indicates the types of H-bonds that are preferentially formed during a jump: H-bonds initially donated from water to the silica layer have highly asymmetric trajectories because half of the final acceptors are water molecules; for this reason, the section of the water–Si trajectory with *t* > 0 is very similar to that of the water–water trajectory. On the other hand, H-bonds donated to the aluminol layer have much more symmetric trajectories, since a greater proportion of the final acceptor is also in this layer.

In this Letter, we have shown that hydrated clays exhibit a variety of nuclear quantum effects, which are rationalized using the angular jump mechanism of hydrogen-bond exchange. The different degrees of quantum effects are found to be a manifestation of the change in confinement that an H-bond experiences at the jump transition state. Future work will focus on understanding the complex jump mechanism in and around clays further, paying attention to the nonmonotonicity in trajectories, as well as the effect of quantum fluctuations at the interface between materials and ionic solutions,^41^ on surfaces at which water molecules dissociate,^42^ and on using more sophisticated potentials^43−45^ or electronic structure theory.

## Supporting Information Available

Files needed to run simulations (ZIP)
Additional information on simulation setup, geometrical criteria, and trajectories for all types of hydrogen-bond jump, and quantum kinetic energy split (PDF)

## References

[ref1] Warne M. R.; Allan N. L.; Cosgrove T. Computer simulation of water molecules at kaolinite and silica surfaces. Phys. Chem. Chem. Phys. 2000, 2, 3663–3668. 10.1039/b004000m.

[ref2] Tunega D.; Gerzabek M. H.; Lischka H. Ab initio molecular dynamics study of a monomolecular water layer on octahedral and tetrahedral kaolinite surfaces. J. Phys. Chem. B 2004, 108, 5930–5936. 10.1021/jp037121g.

[ref3] Hu X. L.; Michaelides A. Water on the hydroxylated (0 0 1) surface of kaolinite: From monomer adsorption to a flat 2D wetting layer. Surf. Sci. 2008, 602, 960–974. 10.1016/j.susc.2007.12.032.

[ref4] Kurapothula P. J. K.; Shepherd S.; Wilkins D. M. Hydrogen Bonding and Nuclear Quantum Effects in Clays. J. Chem. Phys. 2022, 156, 084702 10.1063/5.0083075. 35232185

[ref5] Bhattacharyya K. G.; Gupta S. S. Adsorption of a few heavy metals on natural and modified kaolinite and montmorillonite: A review. Adv. Colloid Interface Sci. 2008, 140, 114 10.1016/j.cis.2007.12.008. 18319190

[ref6] Crasto De Lima F. D.; Miwa R. H.; Miranda C. R. Retention of contaminants Cd and Hg adsorbed and intercalated in aluminosilicate clays: A first principles study. J. Chem. Phys. 2017, 147, 174704 10.1063/1.5009585. 29117701

[ref7] Hatch C. D.; Wiese J. S.; Crane C. C.; Harris K. J.; Kloss H. G.; Baltrusaitis J. Water adsorption on clay minerals as a function of relative humidity: Application of BET and Freundlich adsorption models. Langmuir 2012, 28, 1790–1803. 10.1021/la2042873. 22181675

[ref8] Underwood T.; Erastova V.; Greenwell H. C. Wetting Effects and Molecular Adsorption at Hydrated Kaolinite Clay Mineral Surfaces. J. Phys. Chem. C 2016, 120, 11433–11449. 10.1021/acs.jpcc.6b00187.

[ref9] Pan B.; Yin X.; Iglauer S. A review on clay wettability: From experimental investigations to molecular dynamics simulations. Adv. Colloid Interface Sci. 2020, 285, 102266 10.1016/j.cis.2020.102266. 33011571

[ref10] Hensen E. J.; Smit B. Why clays swell. J. Phys. Chem. B 2002, 106, 12664–12667. 10.1021/jp0264883.

[ref11] Tambach T. J.; Hensen E. J.; Smit B. Molecular simulations of swelling clay minerals. J. Phys. Chem. B 2004, 108, 7586–7596. 10.1021/jp049799h.

[ref12] Laage D.; Hynes J. T. A molecular jump mechanism of water reorientation. Science 2006, 311, 832–5. 10.1126/science.1122154. 16439623

[ref13] Laage D.; Hynes J. T. On the molecular mechanism of water reorientation. J. Phys. Chem. B 2008, 112, 14230–42. 10.1021/jp805217u. 18942871

[ref14] Laage D.; Stirnemann G.; Sterpone F.; Rey R.; Hynes J. T. Reorientation and allied dynamics in water and aqueous solutions. Annu. Rev. Phys. Chem. 2011, 62, 395–416. 10.1146/annurev.physchem.012809.103503. 21219140

[ref15] Cygan R. T.; Liang J. J.; Kalinichev A. G. Molecular models of hydroxide, oxyhydroxide, and clay phases and the development of a general force field. J. Phys. Chem. B 2004, 108, 1255–1266. 10.1021/jp0363287.

[ref16] Greathouse A.; Durkin J. S.; Larentzos J. P.; Cygan R. T. Implementation of a Morse potential to model hydroxyl behavior in phyllosilicates. J. Chem. Phys. 2009, 130, 134713 10.1063/1.3103886. 19355770

[ref17] Pouvreau M.; Greathouse J. A.; Cygan R. T.; Kalinichev A. G. Structure of Hydrated Kaolinite Edge Surfaces: DFT Results and Further Development of the ClayFF Classical Force Field with Metal-O-H Angle Bending Terms. J. Phys. Chem. C 2019, 123, 11628–11638. 10.1021/acs.jpcc.9b00514.

[ref18] Cygan R. T.; Greathouse J. A.; Kalinichev A. G. Advances in Clayff Molecular Simulation of Layered and Nanoporous Materials and Their Aqueous Interfaces. J. Phys. Chem. C 2021, 125, 17573 10.1021/acs.jpcc.1c04600.

[ref19] Habershon S.; Markland T. E.; Manolopoulos D. E. Competing quantum effects in the dynamics of a flexible water model. J. Chem. Phys. 2009, 131, 024501 10.1063/1.3167790. 19603998

[ref20] Parrinello M.; Rahman A. Study of an F center in molten KCl. J. Chem. Phys. 1984, 80, 860 10.1063/1.446740.

[ref21] Rossi M.; Ceriotti M.; Manolopoulos D. E. How to remove the spurious resonances from ring polymer molecular dynamics. J. Chem. Phys. 2014, 140, 234116 10.1063/1.4883861. 24952532

[ref22] Ceriotti M.; More J.; Manolopoulos D. E. i-PI: A Python interface for ab initio path integral molecular dynamics simulations. Comput. Phys. Commun. 2014, 185, 1019–1026. 10.1016/j.cpc.2013.10.027.

[ref23] Kapil V.; et al. i-PI 2.0: A universal force engine for advanced molecular simulations. Comput. Phys. Commun. 2019, 236, 214 10.1016/j.cpc.2018.09.020.

[ref24] Plimpton S. Fast Parallel Algorithms for Short-Range Molecular Dynamics. J. Comput. Phys. 1995, 117, 1 10.1006/jcph.1995.1039.

[ref25] Galicia-Andrés E.; Petrov D.; Gerzabek M. H.; Oostenbrink C.; Tunega D. Polarization Effects in Simulations of Kaolinite-Water Interfaces. Langmuir 2019, 35, 15086–15099. 10.1021/acs.langmuir.9b02945. 31663747 PMC7610636

[ref26] Northrup S. H.; Hynes J. T. The stable states picture of chemical reactions. I. Formulation for rate constants and initial condition effects. J. Chem. Phys. 1980, 73, 2700 10.1063/1.440484.

[ref27] Laage D.; Hynes J. T. On the residence time for water in a solute hydration shell: application to aqueous halide solutions. J. Phys. Chem. B 2008, 112, 7697–7701. 10.1021/jp802033r. 18510295

[ref28] Fogarty A. C.; Duboué-Dijon E.; Laage D.; Thompson W. H. Origins of the non-exponential reorientation dynamics of nanoconfined water. J. Chem. Phys. 2014, 141, 18C523 10.1063/1.4896983. 25399188

[ref29] Laage D.; Thompson W. H. Reorientation dynamics of nanoconfined water: Power-law decay, hydrogen-bond jumps and test of a two-state model. J. Chem. Phys. 2012, 136, 044513 10.1063/1.3679404. 22299897

[ref30] Habershon S. Zero-point energy effects in anion solvation shells. Phys. Chem. Chem. Phys. 2014, 16, 9154–60. 10.1039/c4cp00528g. 24709978

[ref31] Wilkins D. M.; Manolopoulos D. E.; Dang L. X. Nuclear quantum effects in water exchange around lithium and fluoride ions. J. Chem. Phys. 2015, 142, 064509 10.1063/1.4907554. 25681925

[ref32] Kiefer P. M.; Hynes J. T. Kinetic Isotope Effects for Nonadiabatic Proton Transfer Reactions in a Polar Environment. 1. Interpretation of Tunneling Kinetic Isotopic Effects. J. Phys. Chem. A 2004, 108, 11793 10.1021/jp040497p.

[ref33] Laage D.; Hynes J. T. Reorientional dynamics of water molecules in anionic hydration shells. Proc. Natl. Acad. Sci. U. S. A. 2007, 104, 11167–72. 10.1073/pnas.0701699104. 17581877 PMC2040870

[ref34] Boisson J.; Stirnemann G.; Laage D.; Hynes J. T. Water reorientation dynamics in the first hydration shells of F- and I-. Phys. Chem. Chem. Phys. 2011, 13, 19895–901. 10.1039/c1cp21834d. 21915404

[ref35] Fogarty A. C.; Duboué-Dijon E.; Sterpone F.; Hynes J. T.; Laage D. Biomolecular hydration dynamics: a jump model perspective. Chem. Soc. Rev. 2013, 42, 5672–83. 10.1039/c3cs60091b. 23612685

[ref36] Malani A.; Ayappa K. G. Relaxation and jump dynamics of water at the mica interface. J. Chem. Phys. 2012, 136, 194701 10.1063/1.4717710. 22612103

[ref37] Fogarty A. C.; Coudert F.-X.; Boutin A.; Laage D. Reorientational Dynamics of Water Confined in Zeolites. ChemPhysChem 2014, 15, 521 10.1002/cphc.201300928. 24449592

[ref38] Chen M.; Shen W.; Lu X.; Zhu R.; He H.; Zhu J. Jumping Diffusion of Water Intercalated in Layered Double Hydroxides. J. Phys. Chem. C 2016, 120, 12924 10.1021/acs.jpcc.6b04001.

[ref39] Wilkins D. M.; Manolopoulos D. E.; Pipolo S.; Laage D.; Hynes J. T. Nuclear Quantum Effects in Water Reorientation and Hydrogen-Bond Dynamics. J. Phys. Chem. Lett. 2017, 8, 2602 10.1021/acs.jpclett.7b00979. 28530836

[ref40] Li X.-Z.; Walker B.; Michaelides A. Quantum nature of the hydrogen bond. Proc. Natl. Acad. Sci. U. S. A. 2011, 108, 6369–6373. 10.1073/pnas.1016653108.

[ref41] DelloStritto M. J.; Kubicki J. D.; Sofo J. O. Effect of Ions on H-Bond Structure and Dynamics at the Quartz(101)-Water Interface. Langmuir 2016, 32, 11353 10.1021/acs.langmuir.6b01719. 27755876

[ref42] Kumar N.; Kent P. R. C.; Bandura A. V.; Kubicki J. D.; Wesolowski D. J.; Cole D. R.; Sofo J. O. Faster proton transfer dynamics of water on SnO_2_ compared to TiO_2_. J. Chem. Phys. 2011, 134, 044706 10.1063/1.3509386. 21280784

[ref43] Babin V.; Leforestier C.; Paesani F. Development of a “First Principles” Water Potential with Flexible Monomers: Dimer Potential Energy Surface, VRT Spectrum, and Second Virial Coefficient. J. Chem. Theory Comput. 2013, 9, 5395–5403. 10.1021/ct400863t. 26592277

[ref44] Babin V.; Medders G. R.; Paesani F. Development of a “first principles” water potential with flexible monomers. II: Trimer potential energy surface, third virial coefficient, and small clusters. J. Chem. Theory Comput. 2014, 10, 1599–1607. 10.1021/ct500079y. 26580372

[ref45] Medders G. R.; Babin V.; Paesani F. Development of a “first-principles” water potential with flexible monomers. III. Liquid phase properties. J. Chem. Theory Comput. 2014, 10, 2906–2910. 10.1021/ct5004115. 26588266

