# Supplementary material for: Competing Nuclear Quantum Effects and Hydrogen-Bond Jumps in Hydrated Kaolinite
Source: J Phys Chem Lett. 2023 Feb 6;14(6):1542–7. doi: 10.1021/acs.jpclett.2c03896 (PMC9940297; doi:10.1021/acs.jpclett.2c03896)
Supplement: Supplementary file 2 — jz2c03896_si_002.pdf [file jz2c03896_si_002.pdf]

Supporting Information: Competing Nuclear Quantum Effects and Hydrogen Bond Jumps in Hydrated Kaolinite

Pawan K. J. Kurapothula, Sam Shepherd, and David M. Wilkins*

Centre for Quantum Materials and Technology, School of Mathematics and Physics, QueenâĂŹs University Belfast, Belfast BT7 1NN, Northern Ireland, United Kingdom

E-mail: d.wilkins@qub.ac.uk

Contents

1 Simulation Setup S-1

2 Geometrical Criteria for H-Bonding S-3

3 Jump Trajectories Split by Final Acceptor S-3

4 Quantum Kinetic Energy Split by Final Acceptor S-8

Bibliography S-11

References S-11

1 Simulation Setup

The starting point for the simulations of hydrated kaolinite were the optimal kaolinite unit cell found in Ref. S1, in which a 2×2 supercell of kaolinite was simulated with path integral molecular dynamics (PIMD).[S2] The simulation cell was then extended, with a slab of water at its experimental density sandwiched between two of these clay structures. Since atomistic simulations of kaolinite may suffer from artificial electric fields due to the clay sheets acting as parallel-plate capacitors,[S3] two types of system were simulated: one in which both clay layers were oriented with the aluminol layer pointing into the water and one in which the silica layers pointed into the water. Fig. S1 shows the two types of system simulated.

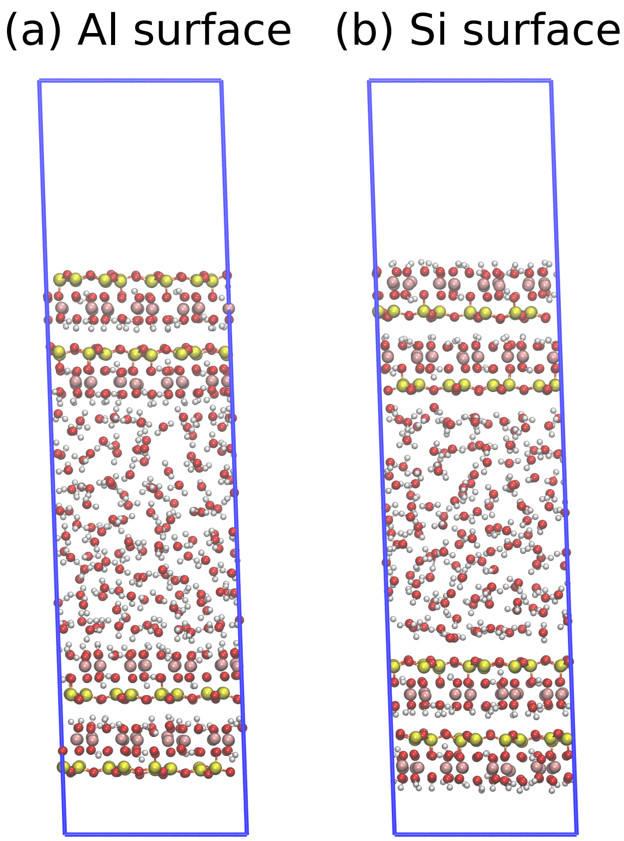

Figure S1: Simulation setup for the two types of system studied in this work: (a) Slab of water between two kaolinite sheets, with the aluminol layer pointing into the water; (b) As (a), but with the silica layer pointing into the water.

Following simulation in the NVT ensemble, a 1 ns production run was carried out for

every system, with frames collected every 5 ps and used to start microcanonical simulations, or in the path integral case thermostatted ring polymer molecular dynamics (TRPMD)[S4] simulations, each of which lasted 100 ps. Example input files for i-PI[S5,S6] and LAMMPS[S7] are included along with the supplementary information.

2 Geometrical Criteria for H-Bonding

As in Ref. S8, we used strict H-bonding criteria to impose stability for the reactant and product states. We used the criteria from a previous study,[S9] in which an O–H group is donating an H-bond to another O atom if the O$\cdots$O distance is less than 2.95 Å, the distance from the donor H to the acceptor O is less than 2.05 Å, and the angle between the O–H vector and the donor O-acceptor O vector is less than 20°.

3 Jump Trajectories Split by Final Acceptor

We show the trajectories close to the transition state for all types of initial acceptor and final acceptor, with the distance from the donor to the initial acceptor ($R_{\text{O-O}_a}$), from the donor to the final acceptor ($R_{\text{O-O}_b}$) and between the two acceptors ($R_{\text{O}_a-\text{O}_b}$, as well as the angle ϕ made by the O_a-O-O_b (initial acceptor-donor-final acceptor oxygen atoms) triplet and the angle θ between the O–H bond vector and the bisector plane of that triplet.[S8]

Figs. S2, S3 and S4 show the trajectories for H-bond jumps, split by initial and final acceptor. For comparison with prior work, e.g. Refs. S8,S9, it should be noted that we are focussing on a much shorter interval of time around the jump transition state than in those references; this is because the focus of the current work is mainly to verify that angular jumps are observed within clays as well as in the water around them; future work will focus on understanding the mechanism in more detail. For this reason, and to minimize the disk space needed to store trajectories, we have stored the frames every 2.5 ps, meaning that the fine-grained dynamics observed for the jump angle cannot be resolved. This tradeoff means that

we were able to investigate the relatively rare jumps involving intralayer H-bonds: Table SI lists the number of H-bond events of each type recorded in our simulations.

Table SI: Number of jump events counted for each type of initial and final H-bond, from classical MD calculations ("Classical Count") and thermostatted ring polymer molecular dynamics calculations ("Quantum Count").

Initial Type	Final Type	Classical Count	Quantum Count
Water-Water	Water-Water	695,772	746,379
Water-Water	Water-Aluminol	12,341	12,929
Water-Aluminol	Water-Aluminol	9,060	10,069
Water-Aluminol	Water-Water	9,001	9,865
Aluminol-Water	Aluminol-Water	10,610	11,151
Aluminol-Water	Intralayer	4,300	4,797
Water-Silica	Water-Silica	5,609	5,480
Water-Silica	Water-Water	16,690	17,474
Interlayer	Interlayer	179	213
Interlayer	Intralayer	2,873	3,163
Intralayer	Intralayer	1,440	1,429
Intralayer	Interlayer	501	313

Figs. S2, S3 and S4 also compare the trajectories from classical MD calculations with those from TRPMD; the effect of quantum fluctuations on the mechanisms of H-bond jumps is seen to be very mild, as was observed in Refs. S9,S10.

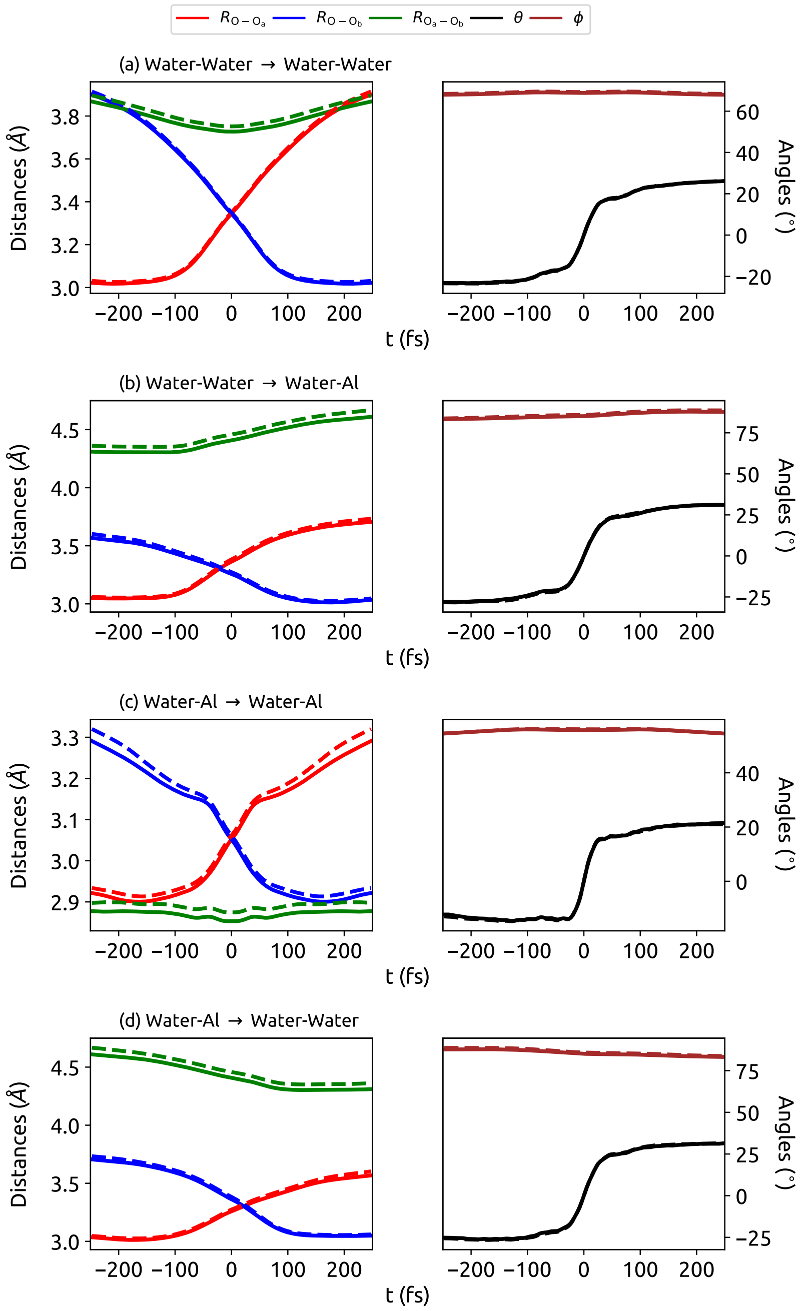

Figure S2: Jump trajectories for hydrogen bonds that are initially between water molecules or from water molecules to aluminol layers of kaolinite (e.g. "Water-Al → Water-Water" refers to a hydrogen bond donated by a water molecule, initially accepted by an aluminol layer and finally accepted by another water molecule). The left-hand panels show distances ($R_{\mathrm{O-O}_a}$ is the distance from the donor O atom to the initial acceptor O atom, $R_{\mathrm{O-O}_b}$ the distance from the donor to the final acceptor and $R_{\mathrm{O}_a-\mathrm{O}_b}$ the distance between the initial and final acceptors) and the right-hand panels angles (ϕ is the O_a-O-O_b angle and θ is the angle between the O–H bond and the bisector of the O_a-O-O_b triplet). Solid lines show the results of classical molecular dynamics, and dashed lines the results of thermostatted ring polymer molecular dynamics.

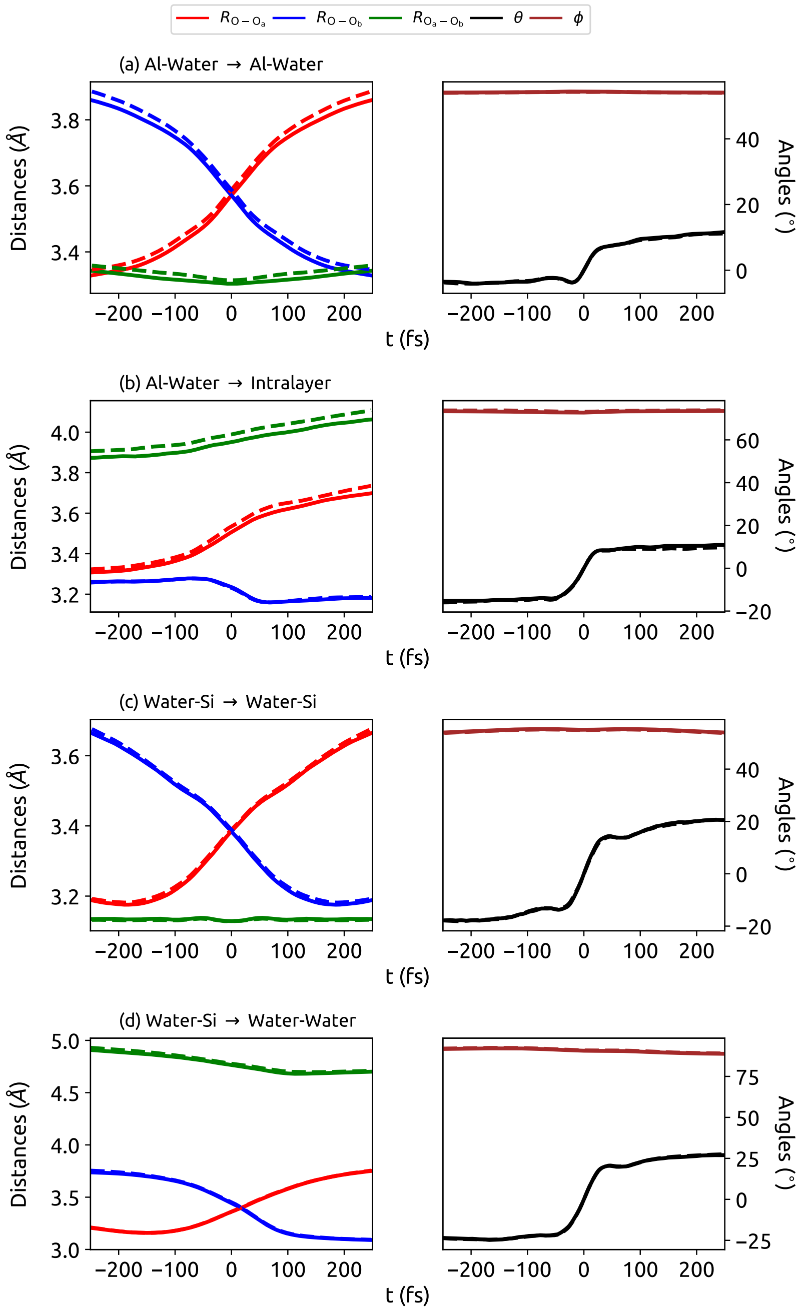

Figure S3: Jump trajectories for hydrogen bonds that are initially donated from aluminol layers of kaolinite to water or by water to kaolinite's silica layers (e.g. "Al-Water → Intralayer" refers to a hydrogen bond donated by an O–H bond in the aluminol layer, initially accepted by a water molecule and finally accepted by another O atom in the aluminol layer). The left-hand panels show distances (R_{O-O_a} is the distance from the donor O atom to the initial acceptor O atom, R_{O-O_b} the distance from the donor to the final acceptor and $R_{O_a-O_b}$ the distance between the initial and final acceptors) and the right-hand panels angles (ϕ is the O_a-O-O_b angle and θ is the angle between the O–H bond and the bisector of the O_a-O-O_b triplet). Solid lines show the results of classical molecular dynamics, and dashed lines the results of thermostatted ring polymer molecular dynamics.

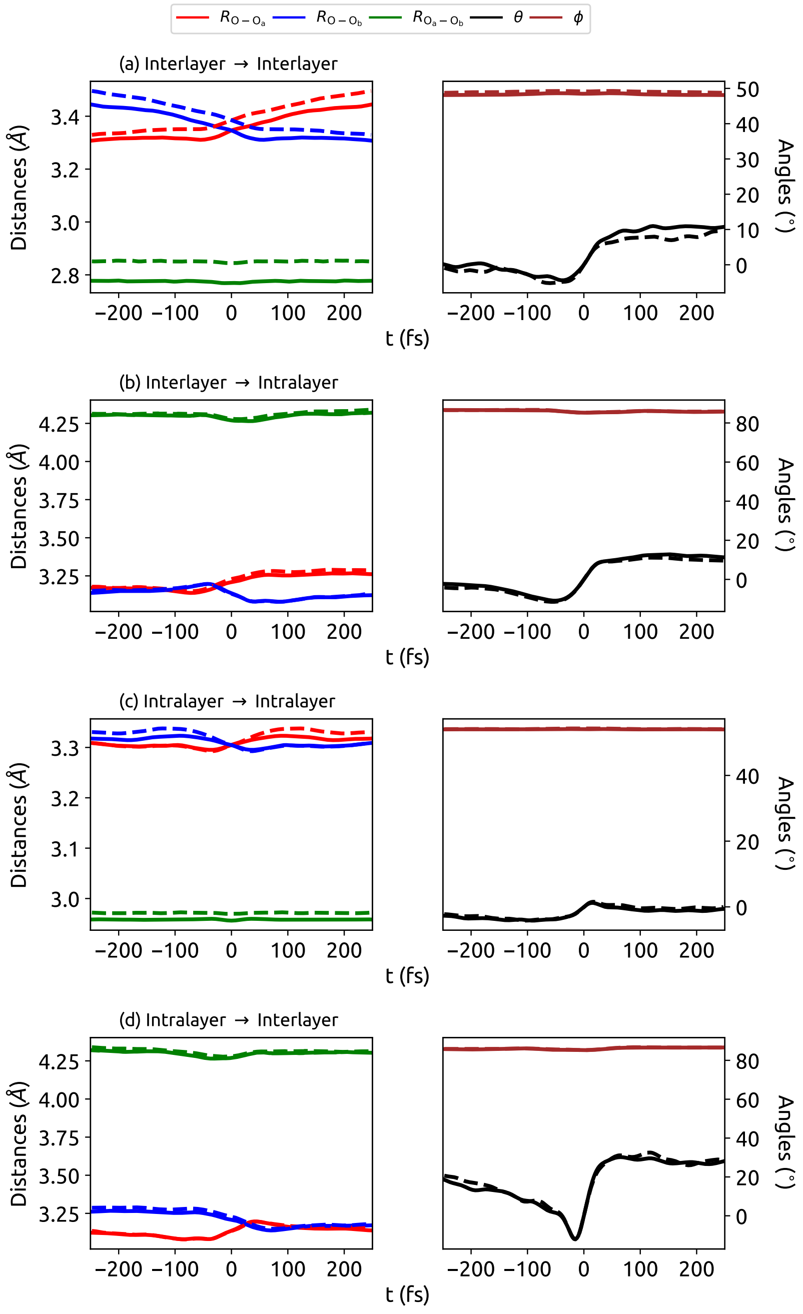

Figure S4: Jump trajectories for hydrogen bonds that are between O atoms in the clay (e.g. "Intralayer → Intralayer" refers to a hydrogen bond initially between two O atoms in the same clay layer, and finally between the same donor and a different acceptor O atom, in the same layer). The left-hand panels show distances ($R_{\mathrm{O-O}_a}$ is the distance from the donor O atom to the initial acceptor O atom, $R_{\mathrm{O-O}_b}$ the distance from the donor to the final acceptor and $R_{\mathrm{O}_a-\mathrm{O}_b}$ the distance between the initial and final acceptors) and the right-hand panels angles (ϕ is the O_a-O-O_b angle and θ is the angle between the O–H bond and the bisector of the O_a-O-O_b triplet). Solid lines show the results of classical molecular dynamics, and dashed lines the results of thermostatted ring polymer molecular dynamics.

4 Quantum Kinetic Energy Split by Final Acceptor

Figs. S5 and S6 show the trajectories for the quantum kinetic energy during H-bond jumps, split by donor, initial and final acceptor. In accord with Table SI, in which the number of jumps from interlayer to intralayer is an order of magnitude larger than from interlayer to interlayer, the kinetic energy trajectories for the former are much less noisy. The trajectories for all H-bonds that begin within the same layer are similarly noisy, although interestingly the jumps from an intralayer to an interlayer H-bond appear to show the perpendicular component $\mathcal{T}_{\perp}$ increasing rather than decreasing on going to the transition state. This implies that the H atom becomes more confined in the perpendicular direction; however, only very few of these jumps were collected, meaning that this contribution to the overall reaction barrier for a jump is relatively small, and that many more jumps would need to be observed to obtain reliable statistics. Given that around ten times as many trajectories were recorded for the reverse type of jump, with interlayer H-bonds becoming intralayer H-bonds, and the corresponding trajectories when time-reversed do not show this trend, it appears likely to be simply an artifact of noise.

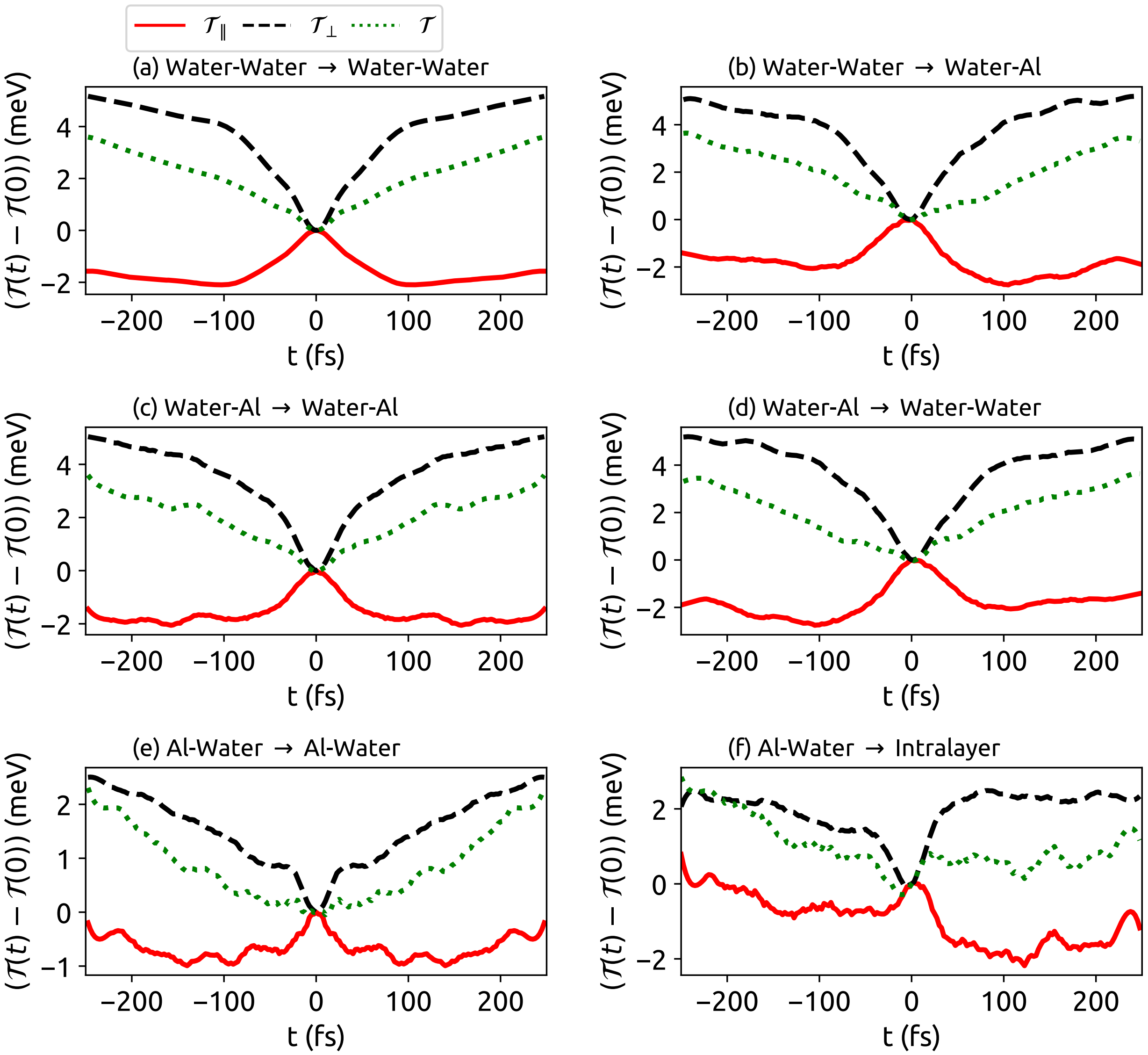

Figure S5: Trajectories for the quantum kinetic energy components in H-bond jumps involving oxygen atoms in water and in aluminol layers. The component of the H atom's kinetic energy tensor parallel ($\mathcal{T}_\parallel$, solid red line) and perpendicular ($\mathcal{T}_\perp$, dashed black line) to the O–H bond are shown, as well as the total kinetic energy $\mathcal{T}$. In all cases, the components are given relative to their value at the jump transition state ($t = 0$).

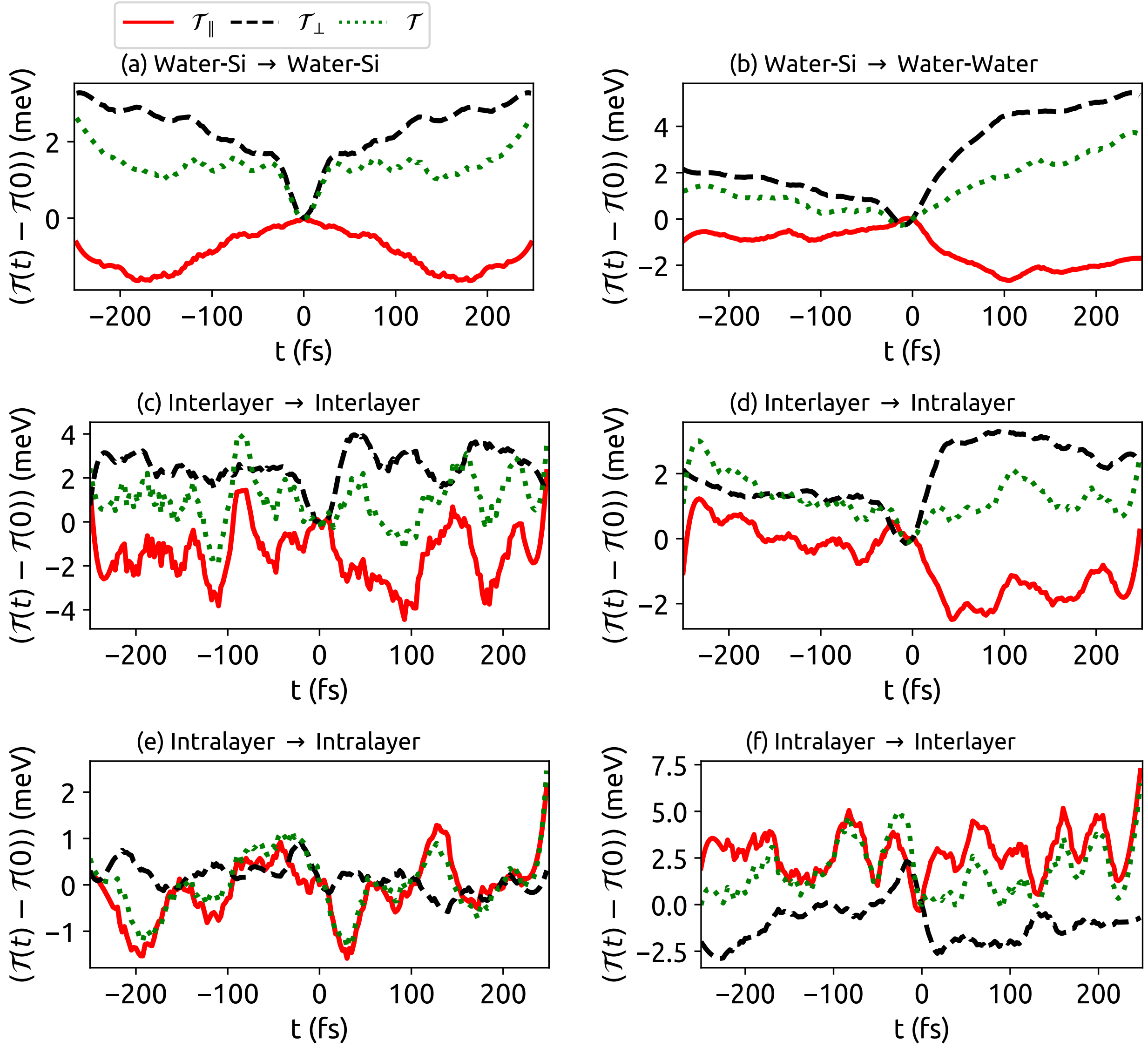

Figure S6: Trajectories for the quantum kinetic energy components in H-bond jumps where the initial hydrogen bond is either donated from water to a clay silica layer, or is internal to the clay. The component of the H atom's kinetic energy tensor parallel ($\mathcal{T}_\parallel$, solid red line) and perpendicular ($\mathcal{T}_\perp$, dashed black line) to the O–H bond are shown, as well as the total kinetic energy $\mathcal{T}$. In all cases, the components are given relative to their value at the jump transition state ($t = 0$).

Bibliography

References

(S1) Kurapothula, P. J. K.; Shepherd, S.; Wilkins, D. M. Hydrogen Bonding and Nuclear Quantum Effects in Clays. *J. Chem. Phys.* **2022**, *156*, 084702.

(S2) Parrinello, M.; Rahman, A. Study of an F center in molten KCl. *J. Chem. Phys.* **1984**, *80*, 860.

(S3) Galicia-Andrés, E.; Petrov, D.; Gerzabek, M. H.; Oostenbrink, C.; Tunega, D. Polarization Effects in Simulations of Kaolinite-Water Interfaces. *Langmuir* **2019**, *35*, 15086–15099.

(S4) Rossi, M.; Ceriotti, M.; Manolopoulos, D. E. How to remove the spurious resonances from ring polymer molecular dynamics. *J. Chem. Phys.* **2014**, *140*, 234116.

(S5) Ceriotti, M.; More, J.; Manolopoulos, D. E. i-PI: A Python interface for ab initio path integral molecular dynamics simulations. *Comput. Phys. Commun.* **2013**, *185*, 1019–1026.

(S6) Kapil, V.; Rossi, M.; Marsalek, O.; Petraglia, R.; Litman, Y.; Spura, T.; Cheng, B.; Cuzzocrea, A.; Meißner, R. H.; Wilkins, D. M.; Juda, P.; Bienvenue, S. P.; Fang, W.; Kessler, J.; Poltavsky, I.; Vandenbrande, S.; Wieme, J.; Corminboeuf, C.; Kühne, T. D.; Manolopoulos, D. E.; Markland, T. E.; Richardson, J. O.; Tkatchenko, A.; Tribello, G. A.; Van Speybroeck, V.; Ceriotti, M. i-PI 2.0: A universal force engine for advanced molecular simulations. *Comput. Phys. Commun.* **2019**, *236*, 214.

(S7) Plimpton, S. Fast Parallel Algorithms for Short-Range Molecular Dynamics. *J. Comp. Phys.* **1995**, *117*, 1.

(S8) Laage, D.; Hynes, J. T. On the residence time for water in a solute hydration shell: application to aqueous halide solutions. *J. Phys. Chem. B* **2008**, *112*, 7697–7701.

(S9) Wilkins, D. M.; Manolopoulos, D. E.; Pipolo, S.; Laage, D.; Hynes, J. T. Nuclear Quantum Effects in Water Reorientation and Hydrogen-Bond Dynamics. *J. Phys. Chem. Lett.* **2017**, *8*, 2602.

(S10) Wilkins, D. M.; Manolopoulos, D. E.; Dang, L. X. Nuclear quantum effects in water exchange around lithium and fluoride ions. *J. Chem. Phys.* **2015**, *142*, 064509.
